# Elucidating the gut microbiome alterations of tribal community of Arunachal Pradesh: perspectives on their lifestyle or food habits

**DOI:** 10.1038/s41598-022-23124-w

**Published:** 2022-10-31

**Authors:** Parijat Hazarika, Indranil Chattopadhyay, Mika Umpo, Yashmin Choudhury, Indu Sharma

**Affiliations:** 1 Department of Microbiology, Assam University, Silchar, 788011 India; 2 Department of Life Sciences, Central University of Tamil Nadu, 610 101, Thiruvarur, India; 3 Department of Microbiology, Tomo Riba Institute of Health and Medical Sciences, Nahrlagun, 791110 India; 4 Department of Biotechnology, Assam University, Silchar, 788011 India

**Keywords:** Microbiology, Molecular biology

## Abstract

Gut microbiota studies of ethnic populations reveal gut microbial biomarkers for therapeutic options and detection of the disease state. The present study aimed to analyze the gut microbiome signatures in thirty individuals from the Adi, Apatani and Nyshi tribes of Arunachal Pradesh (ten in each cohort) by sequencing the V3 and V4 regions of 16S rRNA on the Illumina MiSeq Platform. The gut microbiome was highly predominated by *Firmicutes*, *Actinobacteria*, *Proteobacteria*, and *Bacteroidates* in the three studied tribal groups. At the genus level, significant abundance of *Bifidobacterium*, *Collinsella*, *Bacteroides*, *Prevotella*, *Lactobacillus*, *Streptococcus, Clostridium, Coprococcus, Dorea, Lachnospira, Roseburia, Ruminococcus, Faecalibacterium, Catenibacterium, Eubacterium, Citrobacter* and *Enterobacter* were observed amongst the three tribes. The tribal communities residing in remote areas and following traditional lifestyle had higher gut microbiome diversity with a high prevalence of *Prevotella* and *Collinsella* in the Adi and Nyshi tribes, and *Bifidobacterium* and *Catenibacterium* in the Apatani tribe. Elucidating the gut microbiome of the tribal community of Arunachal Pradesh will add to the knowledge on relationships between microbial communities, dietary food factors, and the overall state of health of humans worldwide.

## Introduction

The human gut microbiota comprises of millions of bacterial cells belonging to several species and modulates many host processes, including metabolism, inflammation, and immune and cellular responses^1^. Other than this, human bone and tissue development can be influenced by human microbiota^2^ and a symbiotic relationship with host can also be maintained^3^. Diet, environment, geography, uses of antibiotics, and host genetics are some factors that could be responsible for making the gut microbiota diverse in structure^4^. Among these varied factors, diet and geography were found to play vital roles in determining the constitution of the human gut microbiome^5–8^. Recently, few studies carried out in rural preindustrialized societies including those still dependant on hunting wild animals, consumption of medicinal plants and populations residing in geographically diverse locations have revealed specific gut microbiota adaptations based on food habits and lifestyle^9–14^. Population based studies may help in understanding the gut microbiota as a target for medical interventions such as the role of fecal microbial transplantations in restoration of the healthy state^15^.

India harbors the second largest population in the world spread across multiple geographical locations with enormous diversity in ethnicity, lifestyle, and dietary habits^16,17^, and few studies on gut microbiome diversity in healthy individuals based on ethnicity, diet, and gender have been previously reported^15,18^^.^ *Firmicutes* are the most common bacteria in healthy Indians' guts, followed by *Bacteroidetes*, *Actinobacteria*, *Proteobacteria*, *Spirochetes, Verrucomicrobia*, and *Fusobacteria*^19^.The geographically isolated tribal populations of India continue to follow a traditional lifestyle, diet and plant-based medicine, and are largely unexposed to modern lifestyles. They therefore provide a unique opportunity to study the human microbiome unaffected by a modern lifestyle. Previous studies have reported the gut microbiota of the ethnic tribes of India to consist mainly of the genera *Prevotella*, *Faecalibacterium*, *Eubacterium*, *Clostridium*, *Blautia*, *Collinsella*, *Ruminococcus*, *Roseburia*, *Dialister*, and *Veillonella*^20–24^. One study reported that the gut microbiota of the Ahom and Bodo ethnic groups who do not consume rice beer mainly consisted of *Faecalibacterium*, *Roseburia*, *Pasteuri*, *Syntrophomonas*, *Acetivibrio*, *Barnesiella*, *Alistipes*, *Oxalobacter*, *Desulfosporosinus*, *Akkerm*ensia, *Anaerobranca* where as *Pediococcus*, *Leptotrichia*, *Fusibacter*, *Streptococcus*, *Nitrosomonas*, *Cystobacter*, *Bavaricoccus*, *Peptococcus*, *Soehngenia* and *Tistrella* while the gut microbiota of rice beer consuming tribal people contained *Fusobacterium* and *Succinivibrio*^21^.

The state of Arunachal Pradesh is located in the foothills of the Himalayas in India’s North-Eastern region, and is considered to be India’s remotest state. It is home to several tribes and sub-tribes, but the gut microbiome signatures of the tribal population from Arunachal Pradesh have not yet been characterized. This study therefore seeks to explore the gut microbiome diversity of healthy individuals belonging to the Adi, Apatani, and Nyshi tribes of Arunachal Pradesh from North East India, by performing 16S metagenomics sequencing of the fecal microbiota of these tribal populations.

## Methods and materials

### Sample size

A total of 30 stool samples were collected through conventional sampling process based on the inclusion and exclusion criteria listed below.

Exclusion criteria were as follows: pregnant women, women attempting to conceive, or nursing mothers; respondents taking any other drugs for long term (including antibiotics during the last 6 months); respondents with acute and chronic gastrointestinal tract diseases such as inflammatory bowel syndrome (IBS), food allergy and lactose intolerance; respondents with one or more co-morbidities such as type 2 diabetes, retinopathy, neuropathy, nephropathy and cardiovascular diseases; respondents with a history of organ transplantation and diseases of the oral cavity. Inclusion criteria were healthy males and females belonging to the age group 20–60 years.

### Study design

Details of the dietary habits, age, and physical status of the respondents belonging to Adi, Apatani and Nyshi tribes of Arunachal Pradesh were recorded. Those individuals who were willing to participate were enrolled in the study (n = 30). Ethics approval for this study was obtained from the Human Ethical Committee, Assam University, Silchar, Assam, India (Ref. NO: IEC/AUS/2019/31/IS, Dated:13/11/2019).

### Sample collection and isolation of DNA

Informed consent was obtained from the enrolled participants using a consent document prior to sample collection and each participant was provided adequate training and instructions regarding the stool collection process. Fecal samples were collected from the volunteers in RNAlater™ (Cat. no. 76104, QIAGEN, and Germany) solution in sterile stool collection tubes and stored at − 80 °C immediately after transportation to the laboratory. DNA was extracted from stool samples using the QIAamp DNA Stool Mini Kit (QIAGEN, Hilden, Germany), following manufacturer’s protocol. DNA concentration was measured using Bio Photometer Plus (Eppendorf, Hamburg, Germany) and the extracted DNA was stored at − 80 °C until further analysis^25,26^.

### Metagenomics analysis using next-generation sequencing (NGS)

16S V3-V4 metagenome library was prepared using V3–V4 region specific primers targeting the Bacterial 16S rRNA gene from stool DNA as per the Illumina protocol^27^. Nextera XT Index Kit (Illumina) was used for sample indexing PCR. The amplicon size of the library was approximately 480 bp and library was purified using AMPure XP beads (Agencourt, Beckman Coulter, USA) and the library was quantified by using Quant-iT™ PicoGreen® dsDNA Reagent (Molecular Probes) at Qubit ® 2.0 Fluorimeter (Life Technologies, USA). The index PCR products were equimolar amounts of each library and were pooled and quantified using DNA High Sensitivity chip at Bioanalyzer (Agilent Technologies) after gel purification. Cluster generation and paired-end sequencing of library mixture was performed using a 500-cycle reagent kit (Illumina MiSeq) as per manufacturer’s instruction^25^.

### Bioinformatics and statistical analysis

Bcl2Fastq tool was used for the demultiplexing of paired-end *V*3–*V*4 reads generated by the Illumina MiSeq platform. Sequence Reads were further assessed based on the quality score by using FastQC (http://www.bioinformatics.babraham.ac.uk/?/projects/fastqc/). Only high quality reads which were joined by Fastq join were considered significant and taken for subsequent analysis^28^. Sequences containing ambiguous bases, homopolymer run exceeding 6, mismatches in primers, or sequence length shorter than 100 bp was removed. The reads were then further analyzed using QIIME (Quantitative Insights Into Microbial Ecology) pipeline^28^. Operational Taxonomic Unit (OTU) were picked at 97% identity against the Green Genes database (v 13.8), and the OTUs were assigned taxonomy based on 97% similarity to the reference sequence^29^. UCLUST method was used to cluster query sequences against a curated chimera free 16S rRNA database (Greengenes v 13.8). All summaries of the taxonomic distributions ranging from phylum to genus were generated from the non-rarefied OTU table generated from this analysis. Generated biom file was then used for further analysis. Clustering was performed on normalized OTU abundances. OTUs representing less than 0.005% of the overall sequence were removed. To characterize diversity across individuals, Alpha-diversity was calculated using Shanon index, inverse Simpson. Beta diversity Principal coordinate analysis (PCoA) was calculated using QIIME. Phylogenetic Investigation of Communities by Reconstruction of Unobserved States (PICRUSt) tool was used to generate biom file for functional analysis. The Kyoto Encyclopedia of Genes and Genomes (KEGG) database (www.kegg.jp) freely available for academic users was used for analysis of the functional compositions of bacterial communities^30–32^. Programs such as ‘adonis’ were used to perform the Permutational Multivariate Analysis of Variance, PERMANOVA (weighted and unweighted unifrac distance matrix), in the R package of ‘vegan’. A heat map was generated using relative abundance values for the top 20 taxa at all taxonomic levels (phylum to genus) across different groups using MEGAN6 (MEGAN_Community_windows-x64_6_24_1.exe). The parametric *G* test was used to determine a statistically significant difference between the abundance of OTUs in different sample groups by comparing the ratio of the observed OTU frequencies in the sample groups to the expected frequencies based on the extrinsic hypothesis. Kruskal–wallis H test was performed in order to compare the groups in the different cohorts based on taxa abundance.

All statistical analyses were performed using R version 3.6.2. Microbial richness, evenness, and diversity were assessed using the R Vegan package. The co-occurrence plot was generated among the taxa for all groups using MEGAN5^33^.

### Ethics approval

The present study was approved by the Human Ethical Committee, Assam University, Silchar, Assam, India (Ref. NO: IEC/AUS/2019/31/IS, Dated:13/11/2019).

### Consent to participate

The samples were collected with the participants’ consent, and the participants duly filled written informed consents.

## Results

### Demographic characteristics of the subjects

Age ranges of 20–60 years were chosen for participants in the current study. There were 16 female participants and 14 male participants. The mean age ± SD of the included respondents from the three tribes were: Adi tribe (39.5 ± 4.3; range 20–60), Apatani tribe (42.5 ± 4.1; range 21–60) and Nyshi tribe (41.9 ± 4.7; range 20–60). The common diet of the Adi and Nyshi tribes consisted mainly of rice with difference in consumption of cereals, millets, leaves, fish and meat. The Apatani tribe mainly consumed boiled rice, boiled vegetables, boiled fish, meat and dairy products in each serving. All three tribes also consumed fermented bamboo shoot, smoked pork and smoked fish (Supplementary Table S1).

### Statistics of sequencing data

**Figure 1:**
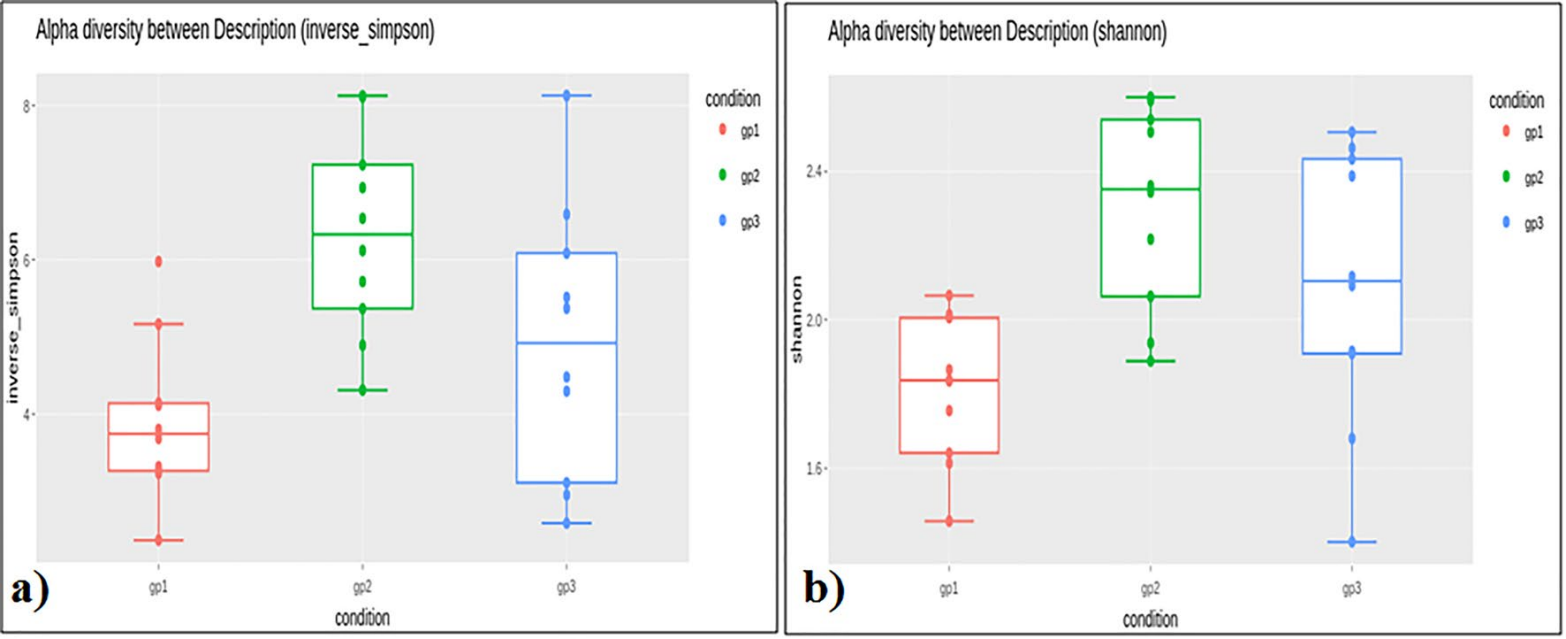
Alpha diversity analysis by using inverse Simpsons diversity indes (**a**) and Shannon index (**b**) (gp1: Adi tribe; gp2: Apatani tribe; gp3: Nyshi tribe).

**Figure 2:**
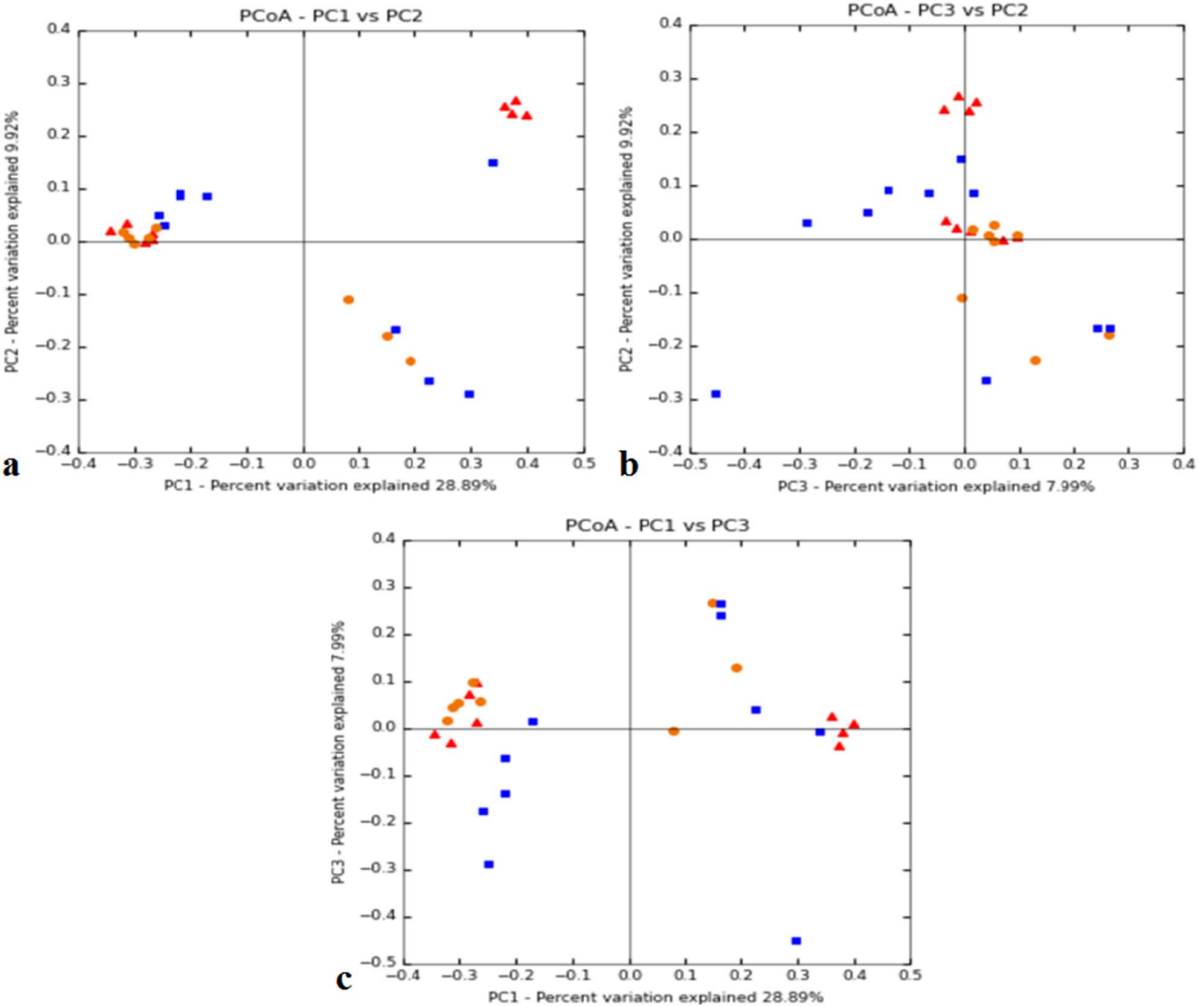
Principal coordinate analysis (PCoA) plot showing the similarity relationships among bacterial community samples from 30 samples divided into three groups (Group_1; Adi tribe, Group_2; Apatani tribe; Group_3; Nyshi tribe).

The number of reads per individual ranged from 44,090 to 70,718 for the Adi tribe; 43,377–112,936 for the Apatani tribe; and, 54,972–109,291 for the Nyshi tribe (Table 1). Alpha diversity assessment of the three studied populations significance difference amongst the tribes using inverse Simpsons diversity index (*p* < 0.004) and Shannon index (*p* < 0.003), respectively (Fig. 1a,b). Kruskal–Wallis rank sum test revealed higher diversity and abundance in Apatani tribe (group 2) than in Adi tribe (group 1) and Nyshi tribe (group 3). Beta diversity was assessed by unweighted unifrac to analyse the similarity of microbiota composition amongst tribes and significantly difference amongst the tribes was observed (R2 = 0.084, *p* = 0.01, Adonis analysis). Principal coordinate analysis (PCoA) indicated that the Adi and Apatani tribes have distinct gut microbiota, while the Nyshi tribe has variable microbiota with their variation in Principal Component (PC1) of 28.89% followed by 9.9% of Principal Component2(PC2) and Principal Component (PC3) of 7.9% (Fig. 2), which may be due to residing in isolated villages with different food habits and lifestyle.

**Table 1:** Read count statistics for adi tribe, Apatani tribe and Nyshi tribe fecal samples of the V3–V4 region; Total OTUs picked—observed species in complete biom.

Sample ID	Total paired end read	Total processed read	Total OTUs picked
ADM01	89,065	58,611	542
ADM02	93,754	50,752	756
ADM03	90,645	64,521	467
ADF04	85,208	52,339	718
ADF05	90,396	60,025	607
ADF06	55,220	51,884	635
ADF07	67,904	46,944	849
ADFO8	66,449	46,868	785
ADM09	93,673	70,718	1084
ADM10	58,788	44,090	875
APF01	73,577	43,377	428
APM02	94,370	60,903	660
APM03	102,694	63,584	1094
APF04	92,753	57,510	1013
APF05	90,743	47,724	1035
APM06	113,363	77,758	917
APM07	60,577	60,886	934
APMO8	101,073	80,086	1501
APF09	118,384	86,433	1497
APF10	198,039	112,936	1930
NM01	87,905	54,972	706
NF02	95,227	61,076	738
NM03	96,199	62,582	709
NM04	93,264	64,328	749
NM05	98,859	59,645	675
NF06	118,374	83,403	1456
NF07	127,532	109,291	1349
NF08	171,503	109,223	1497
NF09	135,881	79,071	1306
NM10	128,279	84,184	824

### Bacterial composition at different taxa of tribal groups

**Figure 3:**
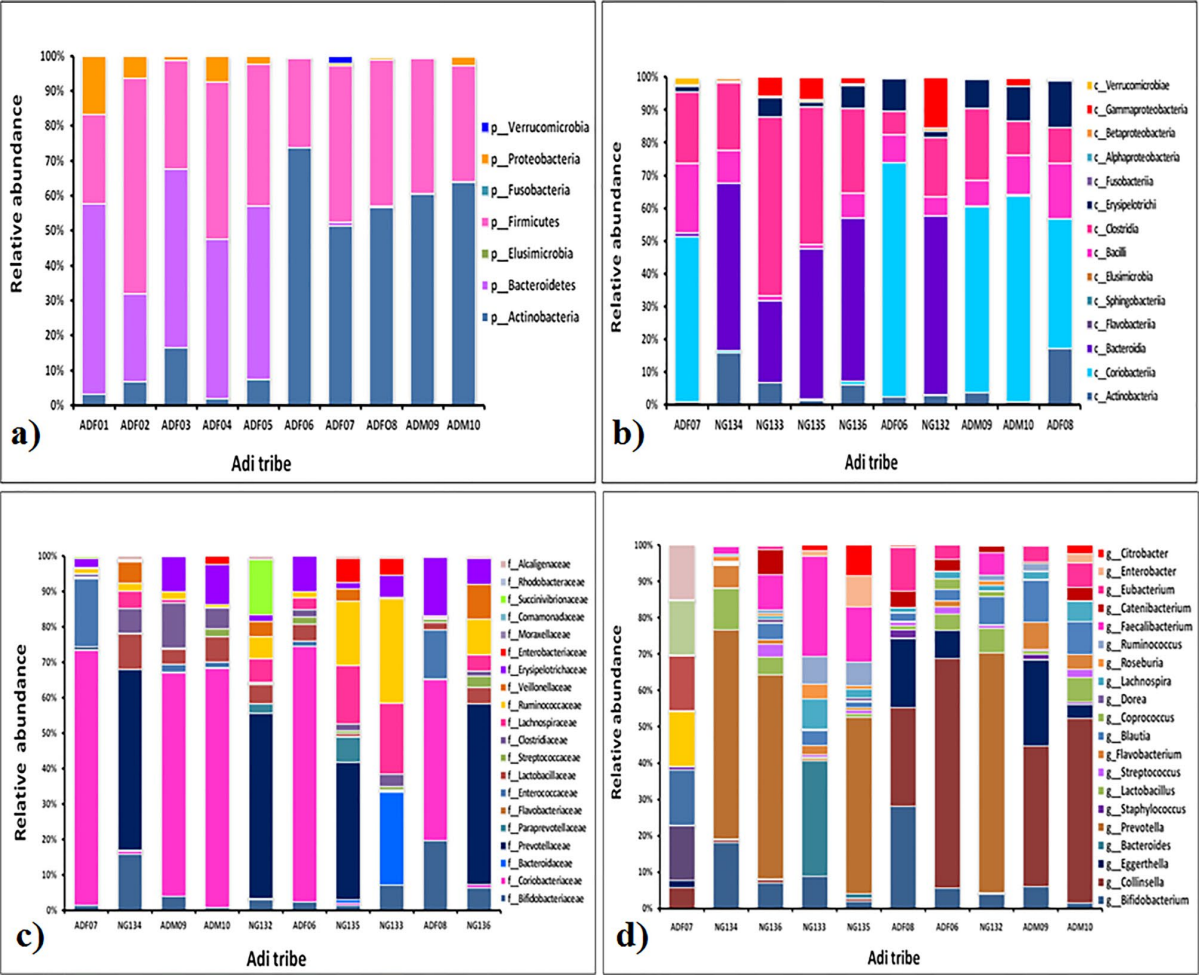
Relative abundance of major taxonomy among the Adi tribe of Arunachal Pradesh at Phylum (**a**), Class level (**b**), family level (**c**), Genus level (**d**).

**Figure 4:**
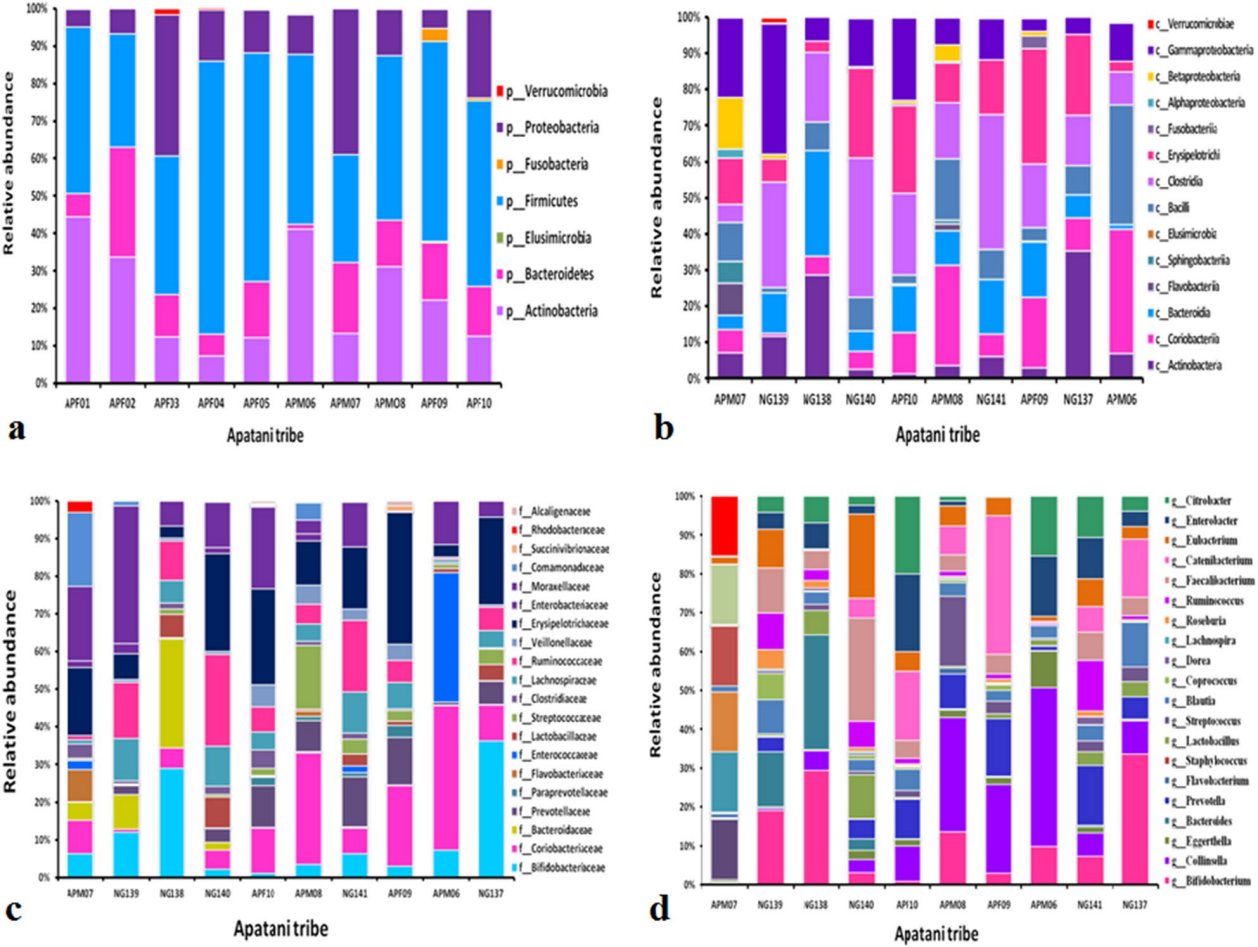
Relative abundance of major taxonomic categories among the Apatani tribe of Arunachal Pradesh at (**a**) Phylum, (**b**) Class, (**c**) family, and (**d**) genus levels.

**Figure 5:**
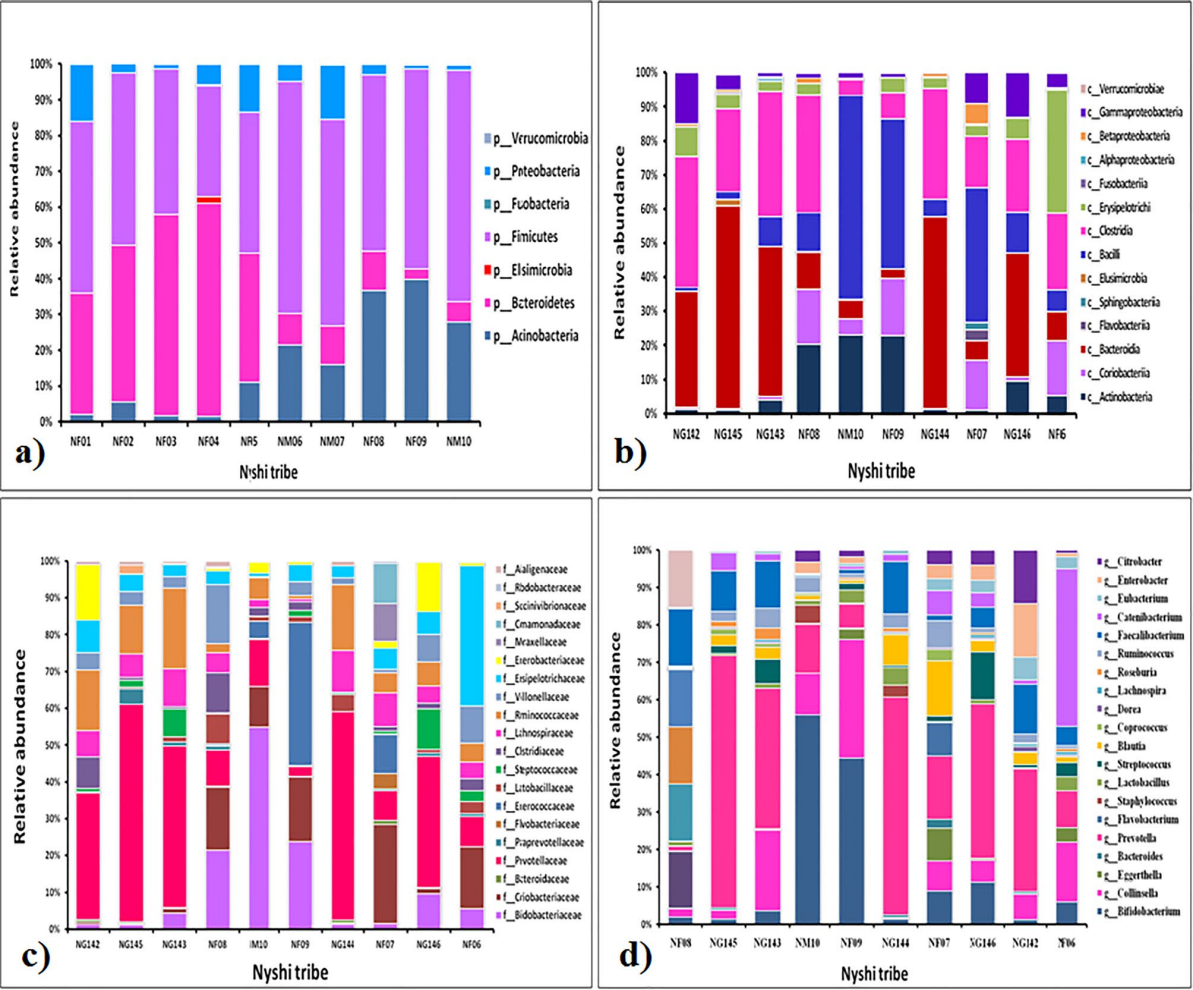
Relative abundance of the major taxonomic categories among the Nyshi tribe of Arunachal Pradesh at (**a**), Phylum, (**b**) Class (**c**) family and (**d**) Genus levels.

Relative abundance of fecal microbiota composition amongst the tribes were categorized at class, phylum, family and genus level. Metagenomics analysis was performed in order to determine the bacterial composition of the GI microbiota of Adi, Apatani and Nyshi tribes of Arunachal Pradesh. It was found that at the phylum level, *Firmicutes, Actinobacteria, Protebacteria, Bacteroidates* showed significant difference in abundance in all the tribes (*p* < 0.05 Kruskal–wallis H test) (Figs. 3a, 4a, 5a). At the class levels, most bacteria amongst tribes belonged to *Actinobacteria, Coriobacteriia, Bacteroidia, Bacilli, Clostridia, Erysipelotrichi, Gammaproteobacteria, Betaproteobacteria,* and *Elusimicrobia* (Figs. 3b, 4b, 5b).

The most abundant families found in the Adi, Apatani and Nyshi tribes of Arunachal Pradesh were *Prevotellaceae, Bifidobacteriaceae, Coriobacteriaceae, Bacteroidaceae, Lactobacillaceae, Streptococcaceae, Lachnospiraceae, Ruminococcaceae, Veillonellaceae, Erysipelotrichaceae, Succinivibrionaceae, Enterobacteriaceae,* and *Rhodobacteraceae* (*p* < 0.05 Kruskal–wallis H test)(Figs. 3c, 4c, 5c). At the genus level, significant abundance of *Bifidobacterium*, *Collinsella*, *Bacteroides*, *Prevotella*, *Lactobacillus*, *Streptococcus, Clostridium, Coprococcus, Dorea, Lachnospira, Roseburia, Ruminococcus, Faecalibacterium, Catenibacterium, Eubacterium, Citrobacter* and *Enterobacter* were observed amongst the three tribes (p < 0.034 Kruskal–wallis H test) (Figs. 3d, 4d, 5d). PERMANOVA analysis also revealed that the different tribal populations have significantly different microbial composition variance at phylum level (F = 3.113, *p* = 0.03), class level (F = 2.801, *p* = 0.024), family level (F = 2.790,*p* = 0.001) and genus levels (F = 2.523, *p* = 0.001).

On comparing amongst tribes, it was observed that *Prevotella* and *Collinsella* were highly prevalent in the Adi (p < 0.032) (Group 1) and Nyshi (p < 0.04) (Group 3) tribes than in the Apatani tribe. On the other hand*, Bifidobacterium* and *Catenibacterium* (*p* < 0.034, *p* < 0.021) were significantly more abundant in the Apatani than the Adi and Nyshi tribes.

For understanding the interactions of different gut bacteria in the different groups, we performed a co-occurrence network analysis by using MEGAN tool. We observed that the genera *Lactococcus*, *Actinomyces*, *Enterococcus*, *Enterococcus* and *Coprococcus* (*p* < 0.01) in the Adi tribe, *Ruminococcus, Enterococcus*, *Streptococcus*, *Coprococcus* and *Staphylococcus* (*p* < 0.004) in Apatani tribe, and *Citrobacter*, *Enterobacter*, *Enterococcus*, *Coprococcus* and *Actinomyces* (*p* < 0.01) in the Nyshi tribe were positively associated with each other (Supplementary Fig. S1). Hierarchical clustering analysis revealed that the Adi, Apatani and Nyshi tribes exhibited distinctly separate clusters at the genus level (Supplementary Fig. S2).

### Differentially altered bacterial abundance in three tribes

While comparing the gut microbiota of the Adi, Apatani and Nyshi tribes using *G*-test analysis at the species level, it was observed that *Coprococcus eutactus* (*p* = 0.01), *Eubacterium biforme* (*p* = 0.001), *Bacteroides uniformis* (*p* = 0.01), *Ruminococcus flavefaciens* (*p* = 0.023), *Bifidobacterium adolescentis* (*p* = 0.001)*, Bacteroides ovatus* (*p* = 0.023), and *Streptococcus anginosus* (*p* = 0.02) were less abundant in the Adi Tribe. The prevalence of *Prevotella copri* (*p* = 0.001)*, Prevotella stercorea* (*p* = 0.001)*, Bacteroides fragilis* (*p* = 0.004)*, Veillonella dispar *(*p* = 0.01)*, Lactobacillus ruminis *(*p* = 0.013), and *Prevotella nanceiensis* (*p* = 0.02) were significantly reduced in the Apatani tribe, while *Bifidobacterium longum*, (*p* = 0.001) and *Dorea formicigenerans *(*p* = 0.017) were significantly elevated in abundance in the Apatani tribe. Similarly, *Prevotella copri* (*p* = 0.001), *Collinsella aerofaciens *(*p* = 0.05) and*Acinetobacter johnsonii* (*p* = 0.05) was found to be significantly reduced in the Nyshi tribe (Table 2).

**Table 2:** Statistically significant different bacterial species in the Adi tribe( Group 1), Apatani tribe(Group 2) and Nyshi tribe(Group 3).

Species	Adi tribe (mean)	Apatani tribe (Mean)	Nyshi tribe (Mean)	Phylum	Abundance status in our present study
*Prevotella stercorea*	115.6	25	21.2	Bacteroidetes	Reduced in Apatani tribe (*p* = 0.001)
*Coprococcus eutactus*	63.8	22.1	70.8	Firmicutes	Reduced in Adi tribe (*p* = 0.01)
*Eubacterium biforme*	12.8	36	2	Firmicutes	Reduced in Adi tribe (*p* = 0.001)
*Bacteroides fragilis*	2122.8	12.4	1251.2	Bacteroidetes	Reduced in Apatani tribe (*p* = 0.004)
*Prevotella copri*	331.6	535.2	14.6	Bacteroidetes	Reduced in Nyshi tribe (*p* = 0.001)
*Bacteroides uniformis*	1.4	181.6	41	Bacteroidetes	Reduced in Adi tribe (*p* = 0.01)
*Ruminococcus flavefaciens*	0.6	38	1.6	Actinobacteria	Reduced in Adi tribe (*p* = 0.023)
*Bifidobacterium adolescentis*	27.2	55	57.6	Actinobacteria	Reduced in Adi tribe (*p* = 0.001)
*Bacteroides ovatus*	2.4	15.4	14	Bacteroidetes	Reduced in Adi tribe (*p* = 0.023)
*Streptococcus anginosus*	2.4	14.2	23.8	Firmicutes	Reduced in Adi tribe (*p* = 0.02)
*Veillonella dispar*	15.8	0.2	23.8	Firmicutes	Reduced in Apatani tribe (*p* = 0.01)
*Collinsella aerofaciens*	11.6	4.1	3.6	Bacteroidetes	Reduced in Nyshi tribe (*p* = 0.05)
*Lactobacillus ruminis*	1.2	0.2	4.1	Firmicutes	Reduced in Apatani tribe (*p* = 0.013)
*Prevotella nanceiensis*	5.6	0.2	7.2	Bacteroidetes	Reduced in Apatani tribe (*p* = 0.02)
*Bifidobacterium longum*	978.8	2852.4	615.2	Actinobacteria	Elevated in Apatani tribe (*p* = 0.001)
*Acinetobacter johnsonii*	2.8	4.4	0	Proteobacteria	Reduced in Nyshi tribe (*p* = 0.05)
*Dorea formicigenerans*	62.9	96.9	70.5	Firmicutes	Elevated in Apatani tribe (*p* = 0.017)

### Functional potential of differentially enriched pathways by PICRUSt analysis

**Figure 6:**
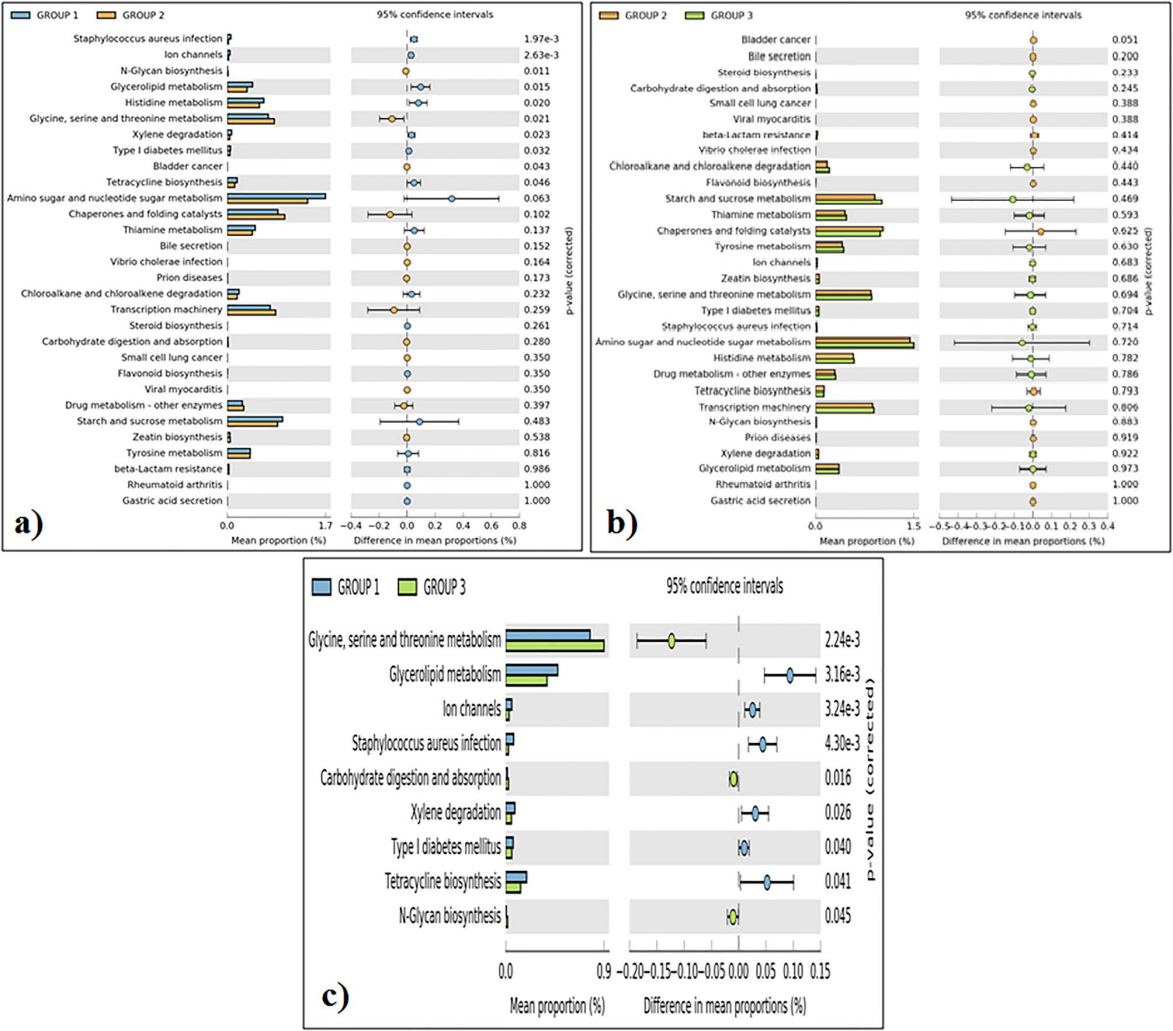
Relative abundances of genes related to different selected function amongst the tribal people (**a**), Adi tribe and Apatani tribe; (**b**) Apatani tribe and Nyshi tribe; (**c**) Adi tribe and Nyshi tribe (Group 1: Adi tribe, Group 2: Apatani tribe, Group3: Nyshi tribe). The significant differences observed between the three groups at 95% confidence level and *p* < 0.05 are reported.

Functional prediction analysis revealed that the gut microbiota of the Adi tribe (group 1) has an abundance of genes relevant to *Staphylococcus aureus* infection, ion channels, glycerolipid metabolism, histidine metabolism, xylene degradation, type I diabetes mellitus and tetracycline biosynthesis (Fig. 6a). The gut microbiota of the Apatani tribe (group 2) has an abundances of genes related to N-glycan biosysthesis, glycine, serine and theronine metabolism, and bladder cancer (Fig. 6b). Similarly, the gut microbiota of the Nyshi tribe (group 3) has high abundances of genes realted to glycine, serine and theronine metabolism, carbohydrate digestion and absorption followed by to N-glycan biosysthesis (Fig. 6c).

## Discussion

Population specific microbiota studies have revealed the distribution of specific microbial communities in their gut. It is very important to understand the role of microbiome in human health^34^. The human gut microbiome composition is unique and stable in each individual living in different geographical locations. Tribal ethnic groups consume different diets and live in isolated villages and environmental conditions from the rest of the world. However, our knowledge on the gut microbiome and genetic diversity of healthy tribals living in such distinct and isolated parts of the state of Arunachal Pradesh in North East India is limited. Therefore, the present study analyzed the gut microbiome composition of healthy respondents belonging to the Adi, Apatani and Nyshi tribes of Arunachal Pradesh. The ethnic tribes of India mostly consume rich food along with dietary fibres including fruits, vegetables and grains. However, the Apatani tribe consumes boiled rice, boiled vegetables, boiled fish, meat and also dairy products in each serving. The common food of the Adi and Nyshi tribes was rice with difference in consumption of cereals, millets, leaves, fish and meat. All three tribes also consume fermented bamboo shoot, smoked pork and smoked fish. Gut microbiota analysis in our study revealed that the gut microbiome of the Adi, Apatani and Nyshi tribes are dominated by the genera *Bifidobacterium, Collinsella, Bacteroides, Prevotella, Lactobacillus, Streptococcus, Clostridium, Coprococcus, Dorea, Lachnospira, Roseburia, Ruminococcus, Faecalibacterium, Oscillospira, Dialister, Catenibacterium, Eubacterium, Citribacter* and *Enterobacter.* In the present study it was observed that *Prevotella*, *Collinsella* were highly prevalent in Adi tribe and Nyshi tribe while lower number was observed in the Apatani tribe. Furthermore, *Bifidobacterium* was observed to be much higher in abundance in the Apatani tribe than the Adi and Nyshi tribe. Presence of *Bifidobacterium* and *Lactobacillus* may be due to consumption of fermented foods, and the high altitude may also influence the composition of human gut microbiota^35^. A recent study^22^ stated that *Bifidobacterium* and *Lactobacillus* exhibited higher abundance in the Lepcha, Bhutia and Nepali populations of Sikkim due to consumption of milk and fermented food products. Interestingly, a study conducted from the urban and rural areas in Delhi found a high abundance of *Prevotella, Lactobacillus, Lachnospira*, and *Roseburia* which are similar to the present study^36^.

Prevalence of bacteria like *Faecalibacterium*, *Lachnospira*, and *Bifidobacterium* contribute anti-inflammatory and antimicrobial activities that act as barrier effect^37,38^. The present study observed a higher number of *Bacteroides* as well as lower number of *proteobacteria* in the Adi and Nyshi tribes than in the Apatani tribe where higher number of *proteobacteria* was observed. Increase of *Bacteroidetes* and proportionally reduced *proteobacteria* may be linked with energy consumption and high animal fat diet consumption^17^. *Prevotellaceae* members belonging to genus such as *Prevotella* which are associated with the food highly rich in carbohydrates derived from plant and fibre in the Indian diet^5,39^. *Prevotella* occurred in high abundance in the gut of Mongolian, Amerindian and Malawian populations due to their carbohydrate rich food habits. In the present study it was observed that *Prevotella copri*, *Eubacterium biforme* were found in higher relative abundance in the Adi, Apatani and Nyshi tribes from Arunachal pradesh which is similar with the previous study^32^. Few studies on *Prevotella copri* in central Indian individuals showed that it was involved in branch-chain amino acids (BCAA) and threonine-independent isoleucine biosynthetic pathway^3^. The present study revealed that at phylum level, *Firmicutes*, *Bacteroidetes*, *Proteobacteria* and *Actinobacteria* are commonly present in all the tribal groups. Similarly, previous study reported that the gut microbiome of Indian communities is dominated by *Firmicutes* followed by *Bacteroidetes, Actinobateria* and *Proteobacteria,* along with *Prevotella, Bifidobacterium*, *Bacteroides, Eubacterium* and *Faecalibacterium* were most prevalent in Indian subjects which is consistent with the present findings^19,40^. Few studies of the Tibetan Malawian, Amerindian and West African populations have also revealed high abundance of *Prevotella*^14,35,39^. Presence of *Bacteroides*, *Ruminococcus,* and *Faecalibacterium* were also observed as the most dominant bacteria in Kerala (South India) populations^23,41^. Similarly, another report from Delhi and Pune Indian populations reported *Actinobacteria*, *Firmicutes*, *Bacteroidetes* and *Proteobacteria* as major phyla and *Prevotella* and *Megasphaera* as the dominant genera^36^. Furthermore, studies from Manipur, Telangana, Assam, and Sikkim have also shown high abundance of *Firmicutes*, *Bacteroidetes* and *Actinobacteria*^22^. While the diet of Indian tribes is mainly dependent on carbohydrate and plant rich food, few reports also describe the abundance of *Prevotella* associated with the animal-based diet^6,19^ and the *Bacteroides* prevalence may be due to consumption of protein rich animal diet^39,42^.The present study observed that healthy tribal people of Adi, Apatani and Nyshi tribes living in Arunachal Pradesh highly prevalent with *Firmicutes* members which is consistent with a previous study^19^.Further, functional genomics analysis revealed that the highly abundant genera encoded several genes with diverse functions. On comparing, it was observed that the microbiota of the Apatani and Nyshi tribes mostly encode genes linked with N-glycan biosysthesis, glycine, serine and theronine metabolism. However, most interestingly, the microbiota of the Adi tribe is predicted to have genes highly linked with *Staphylococcus aureus* infection, ion channels, glycerolipid metabolism, histidine metabolism, xylene degradation, type I diabetes mellitus and tetracycline biosynthesis. Such occurrences in these tribes may be due to far away settlement from industrialization and agricultural pollution^19^.

## Conclusion

In conclusion, the present study elucidated the gut microbiome of the tribal community of Arunachal Pradesh particularly the Adi, Apatani and Nyshi tribes, revealing the presence of beneficial and some harmful bacteria with key genera belonging to *Bifidobacterium*, *Bacteroides*, *Prevotella*, *Lactobacillus*, *Ruminococcus*, *Faecalibacterium* and the phylum *Firmicutes, Actinobacteria, Protebacteria, Bacteroidates*. The dominance of *Firmicutes* and *Bacteroides* in the gut flora of tribal communities of Arunachal Pradesh living in distinct and remote geographical areas was the most significant observation as their presence may be due to rice-based food habits and consumption of traditionally prepared beverages.

## Supplementary Information

Supplementary Information.

## Data Availability

The datasets generated during/or analyzed during the current study are available from the corresponding author on reasonable request.
