# Supplementary material for: Elucidating the gut microbiome alterations of tribal community of Arunachal Pradesh: perspectives on their lifestyle or food habits
Source: Sci Rep. 2022 Oct 31;12:18296. doi: 10.1038/s41598-022-23124-w (PMC9622709; doi:10.1038/s41598-022-23124-w)
Supplement: Supplementary file 1 — Supplementary Information. [file 41598_2022_23124_MOESM1_ESM.pdf]

Elucidating the gut microbiome alterations of tribal community of Arunachal Pradesh - Perspectives on their lifestyle or food habits.

Parijat Hazarika[1], Indranil Chattopadhyay [2], Mika Umpo[3,] Yashmin Choudhury[4] and Indu Sharma[*1]

[1,]Department of Microbiology, Assam University, Silchar-788011, India

[2] Department of Life Sciences, Central University of Tamil Nadu, Thiruvarur-610 101

[3]Department of Microbiology, Tomo Riba Institute of Health and Medical Sciences,

Nahrlagun-791110

[4] Department of Biotechnology, Assam University, Silchar-788011, India

[*1]Corresponding author, Email id: drsharma7652@gmail.com

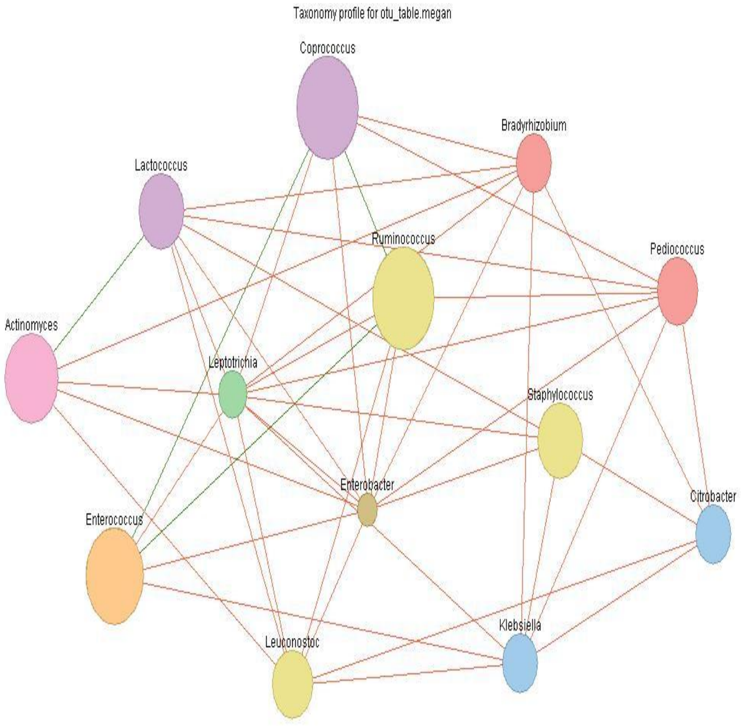
Taxonomy profile for otu_table.megan
Coprococcus
Bradyrhizobium
Lactococcus
Ruminococcus
Pediococcus
Actinomyces
Leptotrichia
Staphylococcus
Enterobacter
Citrobacter
Enterococcus
Klebsiella
Leuconostoc

(a)

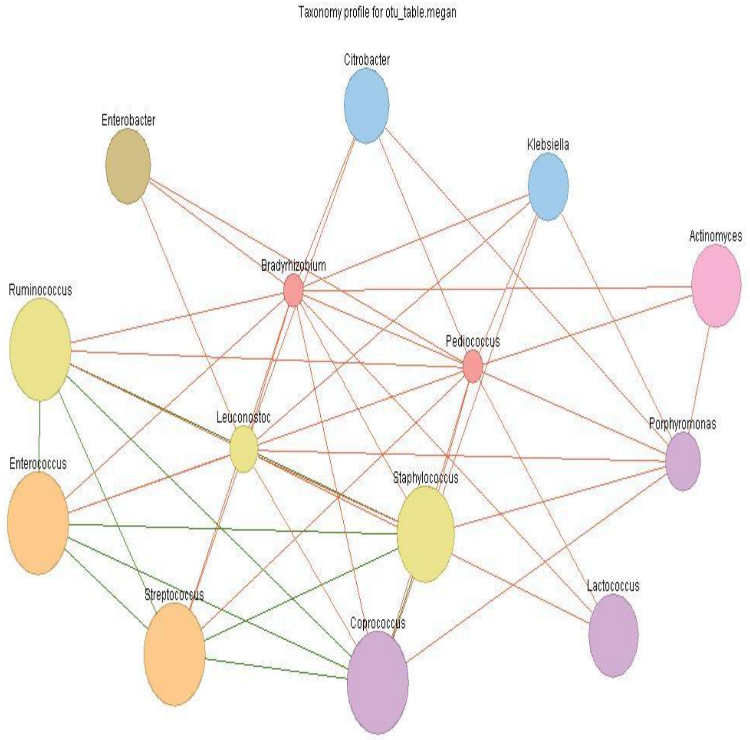
Taxonomy profile for otu_table.megan
Citrobacter
Enterobacter
Klebsiella
Actinomyces
Bradyrhizobium
Ruminococcus
Pediococcus
Leuconostoc
Porphyromonas
Enterococcus
Staphylococcus
Streptococcus
Lactococcus
Coprococcus

(b)

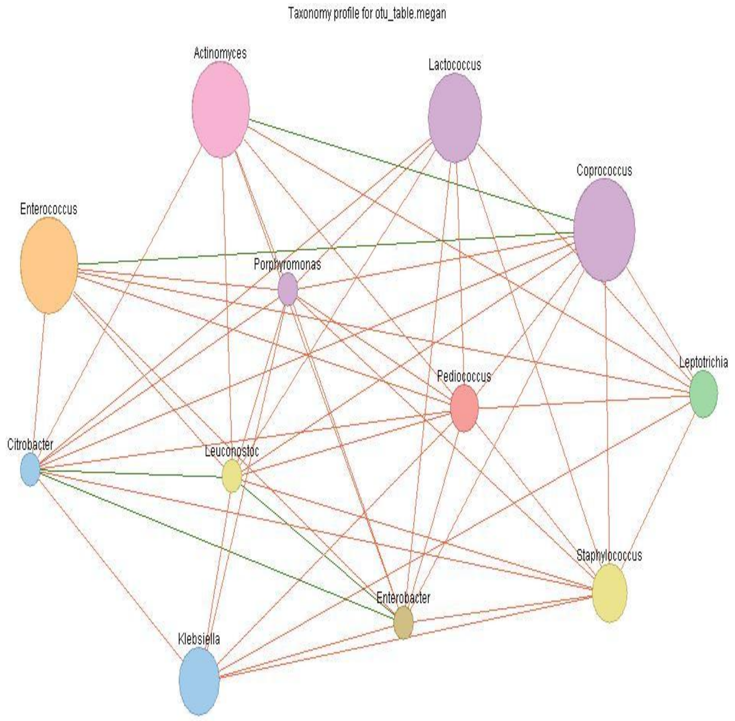

(c)

Figure S1. Co-occurrence networks were constructed on the basis of the relative abundance profiles of fecal microorganisms in the adi tribe(a), Apatani tribe(b), Nyshi tribe (c). Each node represents a genus (Negative: red; positive: green).

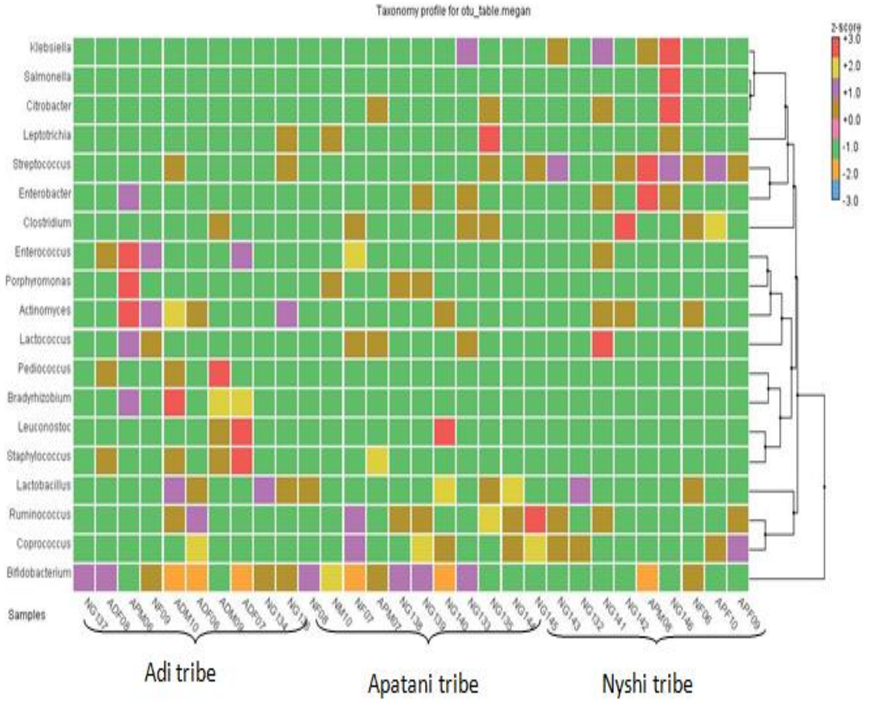

Figure S2. Heat map indicating genus level changes amongst the three groups. MEGAN6 software used to generate (MEGAN_Community_windows-x64_6_24_1.exe).

Table S1. The demographic characteristics of Adi, Apatani and Nyshi tribes of Arunachal Pradesh with their food habits

Sl no	Sample code	Age	Gender	Tribe name	Most common food	Fermented, Smoke & Dried food	Smokers/ Alcoholic
1	ADM01	25	Male	Adi	Rice, Cereal, millets, meat, maize, Lai saag (local name), Dilap (local name), Ongin (local name) etc are the plants used food	Liquid bamboo shoot (ekkung), dry bamboo shoot, smoked fish & meat	Both
2	ADM02	27	Male	Adi	Rice, Cereal, millets, meat, maize, Lai saag (local name), Dilap (local name), Ongin (local name) etc are the plants used food	Liquid bamboo shoot (ekkung), dry bamboo shoot, smoked fish & meat	Apong
3	ADM03	35	Male	Adi	Rice, Cereal, millets, meat, maize, Lai saag (local name), Dilap (local name), Ongin (local name) etc	Liquid bamboo shoot (ekkung), dry bamboo shoot, smoked fish & meat	Both
4	ADF04	32	Female	Adi	Rice, Cereal, millets, meat, maize, Lai saag (local name), Dilap (local name), Ongin (local name) etc are the plants used food	Liquid bamboo shoot (ekkung), dry bamboo shoot, smoked fish & meat	Apong

5	ADF05	40	Female	Adi	Rice , Cereal, millets, meat, maize, Lai saag(local name), Dilap(local name), Ongin(local name) etc are the plants used food	Liquid bamboo shoot (ekkung), dry bamboo shoot, smoked fish & meat	Apong
6	ADF06	51	Female	Adi	Rice, Cereal, millets, meat, maize, Lai saag (local name), Dilap (local name), Ongin (local name) etc are the plants used food	Liquid bamboo shoot (ekkung), dry bamboo shoot, smoked fish & meat	Apong
7	ADF07	59	Female	Adi	Rice, Cereal, millets, meat, maize, Lai saag (local name), Dilap (local name), Ongin (local name) etc are the plants used food	Liquid bamboo shoot (ekkung), dry bamboo shoot, smoked fish & meat	Apong
8	ADFO8	46	Female	Adi	Rice, Cereal, millets, meat, maize, Lai saag (local name), Dilap (local name), Ongin (local name) etc are the plants used food	Liquid bamboo shoot (ekkung), dry bamboo shoot, smoked fish & meat	Apong
9	ADM09	20	Female	Adi	Rice, Cereal, millets, meat, maize, Lai saag (local name), Dilap (local name), Ongin (local name) etc are the plants used food	Liquid bamboo shoot (ekkung), dry bamboo shoot, smoked fish & meat	Apong
10	ADM10	60	Female	Adi	Rice, Cereal, millets, meat, maize, Lai saag (local name), Dilap (local name), Ongin (local name) etc are the plants used food	Liquid bamboo shoot (ekkung), dry bamboo shoot, smoked fish & meat	Both
11	APF01	39	Male	Apatani	Boiled Rice, boiled green leafy boiled vegetables, boiled meat, mithun, boiled fish	Fermented bamboo shoot, smoked fish & Pika pila (pickle made by using bamboo shoot with pork fat) meat	Apong
12	APM02	23	Male	Apatani	Boiled Rice, boiled green leafy boiled vegetables, boiled meat, mithun, boiled fish	Fermented bamboo shoot, smoked fish & Pika pila(pickle made by using bamboo shoot with pork fat) meat	Apong
13	APM03	32	Male	Apatani	Boiled Rice, boiled green leafy boiled vegetables, boiled meat, mithun, boiled fish	Fermented bamboo shoot, smoked fish & Pika pila (pickle made by using bamboo shoot with pork fat) meat	Apong
14	APF04	50	Female	Apatani	Boiled Rice, boiled green leafy boiled vegetables, boiled meat, mithun, boiled fish	Fermented bamboo shoot, smoked fish & Pika pila (pickle made by using bamboo shoot with pork fat) meat	Tobacco
15	APF05	45	Female	Apatani	Boiled Rice, boiled green	Fermented bamboo	Tobacco

					leafy boiled vegetables, boiled meat, mithun, boiled, fish	shoot, smoked fish & Pika pila (pickle made by using bamboo shoot with pork fat) meat	
16	APM06	21	Male	Apatani	Boiled Rice, boiled green leafy boiled vegetables, boiled meat, mithun, boiled, fish, dairy product	Fermented bamboo shoot, smoked fish & Pika pila (pickle made by using bamboo shoot with pork fat) meat	Apong
17	APM07	49	Male	Apatani	Boiled Rice, boiled green leafy boiled vegetables, boiled meat, mithun, boiled, fish, dairy products	Fermented bamboo shoot, smoked fish & Pika pila (pickle made by using bamboo shoot with pork fat) meat	Apong
18	APMO8	60	Male	Apatani	Boiled Rice, boiled green leafy boiled vegetables, boiled meat, mithun, boiled, fish, dairy products	Fermented bamboo shoot, smoked fish & Pika pila (pickle made by using bamboo shoot with pork fat) meat	Apong
19	APF09	51	Female	Apatani	Boiled Rice, boiled green leafy boiled vegetables, boiled meat, mithun, boiled, fish, dairy products	Fermented bamboo shoot, smoked fish & Pika pila (pickle made by using bamboo shoot with pork fat) meat	Apong
20	APF10	55	Female	Apatani	Boiled Rice, boiled green leafy boiled vegetables, boiled meat, mithun, boiled, fish, dairy products	Fermented bamboo shoot, smoked fish & Pika pila (pickle made by using bamboo shoot with pork fat) meat	Apong
21	NM01	48	Male	Nyshi	Rice, green leafy vegetables, meat, fish,	Fermented bamboo shoot, soya bean, smoked fish & meat	Upo or Apong (made of rice) and polin (made of millet)
22	NF02	20	Female	Nyshi	Rice, green leafy vegetables, meat, fish	Fermented bamboo shoot, soya bean, smoked fish & meat	Both
23	NM03	51	Male	Nyshi	Rice, green leafy vegetables, meat, fish	Fermented bamboo shoot, soya bean, smoked fish & meat	Upo or Apong (made of rice) and polin (millet)
24	NM04	23	Male	Nyshi	Rice, millet, green leafy vegetables, meat, fish	Fermented bamboo shoot, soya bean, smoked fish & meat	Both

25	NM05	37	Male	Nyshi	Rice, green leafy vegetables, meat, fish	Fermented bamboo shoot, soya bean, smoked fish & meat	Upo or Apong (made of rice) and polin (millet)
26	NF06	54	Female	Nyshi	Rice, green leafy vegetables, meat, fish	Fermented bamboo shoot, soya bean, smoked fish & meat	Upo or Apong (made of rice) and polin (made of millet)
27	NF07	22	Female	Nyshi	Rice, green leafy vegetables, meat, fish	Fermented bamboo shoot, soya bean, smoked fish & meat	Upo or Apong(made of rice) and polin (made of millet)
28	NF08	60	Female	Nyshi	Rice, green leafy vegetables, meat, fish	Fermented bamboo shoot, soya bean, smoked fish & meat	Upo or Apong (made of rice) and polin (made of millet)
28	NF09	60	Female	Nyshi	Rice, green leafy vegetables, meat, fish	Fermented bamboo shoot, soya bean, smoked fish & meat	Upo or Apong (made of rice) and polin (made of millet)
30	NM10	44	Male	Nyshi	Rice, green leafy vegetables, meat, fish	Fermented bamboo shoot, soya bean, smoked fish & meat	Upo or Apong (made of rice) and polin (made of millet)
